# 2233. Burden of Bacterial Antimicrobial Resistance in United States in 2019: A Systematic Analysis

**DOI:** 10.1093/ofid/ofad500.1855

**Published:** 2023-11-27

**Authors:** Vishrant Prafulchandra Amin, Maulik Dhanani, Juhi Patel, Arushi Dhawan, Gangannapalle Mahesh, Venkata Sai Harshabhargav Chenna, Sumit Kyada, Anushka Dekhne, Hardik Dineshbhai Desai

**Affiliations:** GMERS Medical College, Valsad, MILLTOWN, New Jersey; Southwestern University, School of Medicine, Cebu, Cebu, Philippines; GMERS Medical College, Valsad, MILLTOWN, New Jersey; Punjab Institute of Medical Sciences,garha road,, Jalandhar, Punjab, India; UNIVERSITY OF PERPETUAL HELP SYSTEM DALTA, Las pinas, Laguna, Philippines; University of perpetual help system dalta, Las pinas, Laguna, Philippines; GMERS Medical College, Valsad, MILLTOWN, New Jersey; American University of Antigua, St Johns, Antigua, Osbourn, Saint John, Antigua and Barbuda; Gujarat Adani Institute of Medical Sciences, Affiliated K.S.K.V University, Ahmedabad, Gujarat, India

## Abstract

**Background:**

The burden of AMR in the United States (US) is significant. According to the Centers for Disease Control and Prevention (CDC), at least 2.8 million people in the United States acquire antibiotic-resistant infections each year, and more than 35,000 people die as a result.

**Methods:**

Using the Global Burden of Disease, Antimicrobial resistance (AMR) methodology, data were obtained on deaths and DALYs attributable to and associated with bacterial AMR for 23 pathogens in the US in 2019.

**Results:**

In the US, there were 60,813 deaths (95% uncertainty interval [UI]:32,520-102,231) associated with and 14,987 deaths (95% UI:7,712-25,156) attributable to bacterial AMR in blood stream infection highest in 2019. Staphylococcus aureus (61,011) and E Coli (41,656) accounted for the majority of deaths attributable to and associated with AMR in 2019, and resistance was high among multiple types of antibiotic class. There is a significant burden of AMR in Staphylococcus aureus, with up to 50% of the resistance attributed to macrolides, 38% attributable to fluoroquinolones.

Deaths associated and attributable to bacterial antimicrobial resistance by pathogen in USA, in 2019

**Figure:**
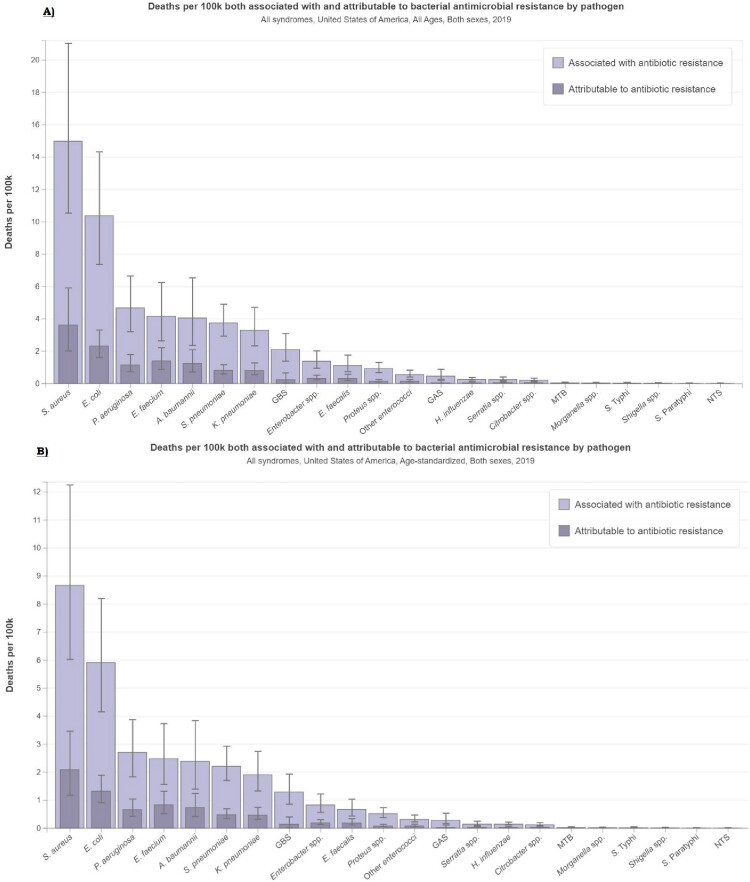

A) All age-counts B) Age-standardized Rate

Deaths Associated with and attributable to bacterial Antimicrobial resistance by syndrome in USA, in 2019

**Figure:**
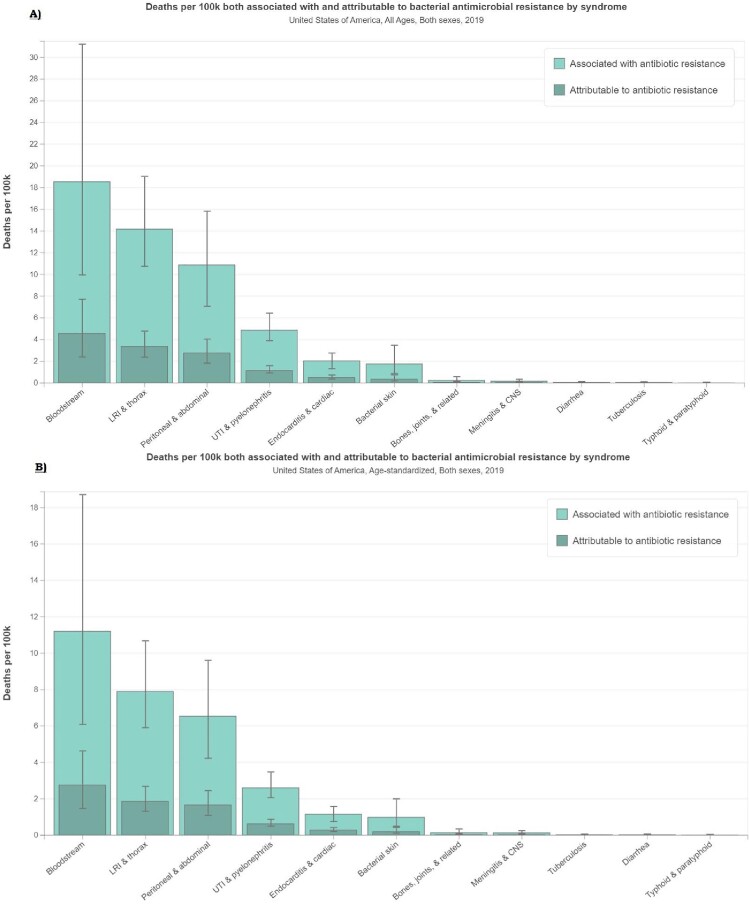

A) All age counts B) Age-Standardized Rate

DALYs attributable to and associated with bacterial antimicrobial resistance in USA, in 2019

**Figure:**
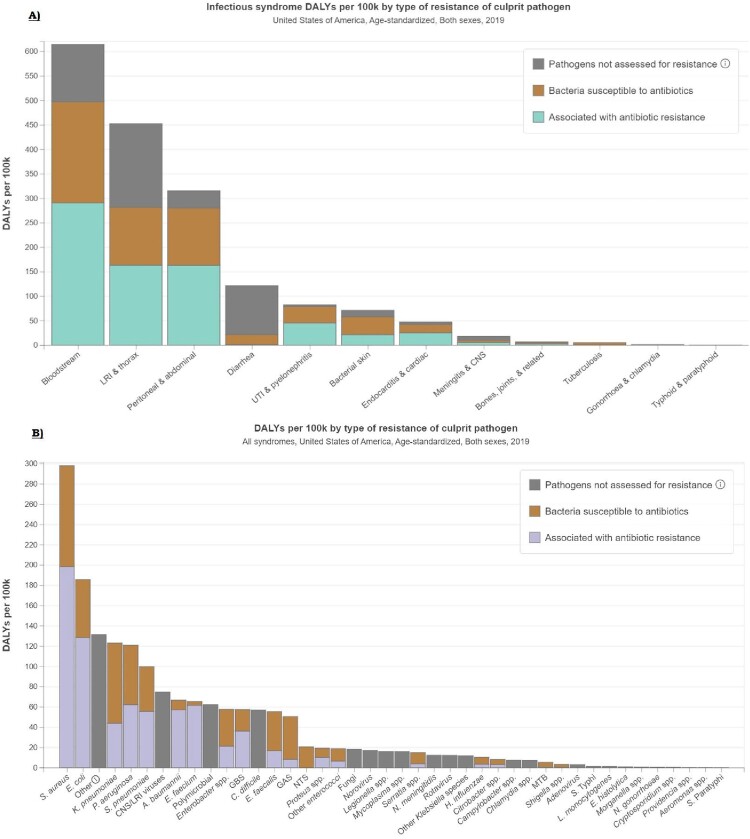

A) Age-standardized DALY by Infectious Syndrome B) Age-Standardized DALY by Pathogen

**Conclusion:**

AMR is a serious burden in the United States, with millions of people acquiring antibiotic-resistant infections each year and tens of thousands dying as a result. Preventing the spread of AMR requires a multifaceted approach that includes improved infection control practices, more judicious use of antimicrobial drugs, increased surveillance and monitoring of resistance patterns, and the development of new antibiotics and other treatment options.

**Disclosures:**

**All Authors**: No reported disclosures

